# Trends in Intracranial and Cerebral Volumes of Framingham Heart Study Participants Born 1930 to 1970

**DOI:** 10.1001/jamaneurol.2024.0469

**Published:** 2024-03-25

**Authors:** Charles DeCarli, Pauline Maillard, Matthew P. Pase, Alexa S. Beiser, Daniel Kojis, Claudia L. Satizabal, Jayandra J. Himali, Hugo J. Aparicio, Evan Fletcher, Sudha Seshadri

**Affiliations:** 1 Department of Neurology & Imaging of Dementia and Aging Laboratory, University of California Davis, Sacramento, California; 2 Framingham Heart Study, Framingham, Massachusetts; 3 Turner Institute for Brain and Mental Health, Monash University, Melbourne, Australia; 4 Harvard T.H. Chan School of Public Health, Harvard University, Boston, Massachusetts; 5 Department of Biostatistics, Boston University School of Public Health, Boston, Massachusetts; 6 Department of Neurology, Boston University Chonbanian and Avedisian School of Medicine, Boston, Massachusetts; 7 The Glenn Biggs Institute for Alzheimer’s and Neurodegenerative Diseases, University of Texas Health Sciences Center, San Antonio; 8 Department of Population Health Sciences, UT Health San Antonio, San Antonio, Texas

## Abstract

**Importance:**

Human brain development and maintenance is under both genetic and environmental influences that likely affect later-life dementia risk.

**Objective:**

To examine environmental influences by testing whether time-dependent secular differences occurred in cranial and brain volumes and cortical thickness over birth decades spanning 1930 to 1970.

**Design, Setting, and Participants:**

This cross-sectional study used data from the community-based Framingham Heart Study cohort for participants born in the decades 1930 to 1970. Participants did not have dementia or history of stroke and had magnetic resonance imaging (MRI) obtained from March 18, 1999, to November 15, 2019. The final analysis dataset was created in October 2023.

**Exposure:**

Years of birth ranging from 1925 to 1968.

**Main Measures:**

Cross-sectional analysis of intracranial, cortical gray matter, white matter, and hippocampal volumes as well as cortical surface area and cortical thickness. The secular measure was the decade in which the participant was born. Covariates included age at MRI and sex.

**Results:**

The main study cohort consisted of 3226 participants with a mean (SD) age of 57.7 (7.8) years at the time of their MRI. A total of 1706 participants were female (53%) and 1520 (47%) were male. The birth decades ranged from the 1930s to 1970s. Significant trends for larger intracranial, hippocampal, and white matter volumes and cortical surface area were associated with progressive birth decades. Comparing the 1930s birth decade to the 1970s accounted for a 6.6% greater volume (1234 mL; 95% CI, 1220-1248, vs 1321 mL; 95% CI, 1301-1341) for ICV, 7.7% greater volume (441.9 mL; 95% CI, 435.2-448.5, vs 476.3 mL; 95% CI, 467.0-485.7) for white matter, 5.7% greater value (6.51 mL; 95% CI, 6.42-6.60, vs 6.89 mL; 95% CI, 6.77-7.02) for hippocampal volume, and a 14.9% greater value (1933 cm^2^; 95% CI, 1908-1959, vs 2222 cm^2^; 95% CI, 2186-2259) for cortical surface area. Repeat analysis applied to a subgroup of 1145 individuals of similar age range born in the 1940s (mean [SD] age, 60.0 [2.8] years) and 1950s (mean [SD] age, 59.0 [2.8] years) resulted in similar findings.

**Conclusion and Relevance:**

In this study, secular trends for larger brain volumes suggested improved brain development among individuals born between 1930 and 1970. Early life environmental influences may explain these results and contribute to the declining dementia incidence previously reported in the Framingham Heart Study cohort.

## Key Points

**Question:**

Are there secular differences in cranial and brain size?

**Findings:**

This cross-sectional study found secular differences of larger cranial and brain volumes among individuals without dementia and history of stroke who were born during the decades spanning 1930 to 1970.

**Meaning:**

Secular differences in early life environment are leading to greater brain development that may reduce the risk for late-life dementia.

## Teaser

This cross-sectional study examines data from participants of the Framingham Heart Study who had magnetic resonance imaging to determine differences in cranial and brain size across 5 decades of birth.

## Introduction

The overall health of the US population has improved dramatically over the last 100 years,^1^ although health disparities persist.^2^ Individuals are also living longer, resulting in an increasing percentage of the population at risk for Alzheimer disease and related dementias. However, recent data,^3,4^ including data from the Framingham Heart Study (FHS),^5^ indicate that dementia incidence may be declining. While such factors as greater educational achievement^6^ and medical management of vascular risk factors^7,8^ may explain part of this effect, early life environmental differences also likely contribute.^6^

Initiated in 1948, the FHS consists of multiple generations of participants across more than 80 years’ difference in birth dates, making it ideal for investigating secular trends in cardiovascular and brain health.^7,9^ The Framingham magnetic resonance imaging (MRI) study, initiated in 1999, has imaged 5145 unique individuals across all 3 cohorts, enabling age-specific comparisons of secular trends. We hypothesized that secular trends in early life health, as observed in the general US population,^1^ would be accompanied by better brain development, which would be represented by larger intracranial and regional brain volumes.

## Methods

All participants provided written informed consent. The consent form and study protocol were approved by the institutional review board at Boston University Medical Center. Reporting for this study followed relevant guidelines for an original investigation involving epidemiological assessment.

### Study Design

The general design and demographics of the FHS have been previously described.^9,10,11^ In brief, the FHS is a community-based population study of more than 15 000 individuals from the town of Framingham, Massachusetts, spanning more than 75 years of observation. The original cohort of the FHS included 5209 participants who were enrolled into the study in 1948. At enrollment, the mean age was 44 years (range, 28-62 years), 2865 (55%) were female, and 2344 (45%) were male; the majority were White and of middle socioeconomic class. The offspring cohort included 5124 offspring of the original cohort and their spouses enrolled in 1971. At enrollment, the mean age was 36 years (range, 25-70 years), 2664 (52%) were female, and 2460 (48%) were male. The third-generation cohort included 4095 children of the offspring cohort enrolled in 2002. At enrollment, the mean age was 41 years (range, 25-70 years), 2170 (53%) were female, and 1925 (47%) were male.

In addition to repeated examination cycles, surviving participants are under surveillance for incident events, such as myocardial infarction, stroke, and dementia. As part of multiple large ancillary studies on brain structure and cognitive function starting in March 1999, FHS participants were recruited to undergo MRI of the brain and a neuropsychological test battery as previously described.^12^

To examine secular trends in brain volume, only initial visits where MRI was performed were selected. This resulted in a cohort of 5060 individuals with complete MRI, height, and demographic data born between 1902 and 1985. MRI was performed from March 18, 1999, to November 15, 2019, on a total of 12 different MRI machines. However, most participants were scanned on only 2 different Siemens machines. To reduce the effect of machine variability on MRI measures, a subgroup of 4647 individuals imaged with these 2 scanners was selected. To assure maximal MRI age overlap among decade of birth cohorts, the study sample was further restricted to those aged 45 to 70 years at the time of their MRI, resulting in 3407 individuals for these analyses. Of 3407 participants, 181 were excluded because they were missing an assessment or had prevalent dementia, stroke, or other significant neurological disorder (eg, multiple sclerosis) at the time of the MRI.

### Outcomes

Height measurement in inches was obtained at the first FHS visit for each participant. This measurement occurred at a mean (SD) age of 38.6 (8.0) years, which means it was not affected by later-life skeletal degeneration.

Quantifications of MRI measures were obtained from high-resolution 3-dimensional (3-D) T1-weighted MP RAGE images using either a Siemens Magneton Expert or Siemens Avanto machine to obtain intracranial, cortical gray matter, cerebral white matter, hippocampal volume, cortical surface area, and cortical thickness measures. Quantification included automatic removal of nonbrain elements from the 3-D T1 image volume using a robust and accurate convolutional neural network method relatively insensitive to machine type or field strength.^13^ Intracranial volume (ICV) was defined as the cranial vault above the tentorium. Image intensity correction was used to remove B1 inhomogeneity effects,^14^ and segmentation of the image into 3 tissue classes used an algorithm optimized to improve precision of tissue segmentation (gray, white, cerebrospinal fluid^14,15^). Additionally, hippocampal analyses were performed using an atlas-based diffeomorphic approach^16^ with the minor modification of label refinement. Cortical thickness was measured using the DireCT method.^17^ Cortical surface area was calculated using 3-D cortical surface mesh reconstructions employing marching cubes,^18^ a standard algorithm in computer graphics that has been used extensively in brain image analyses.^19^ Using this approach, we defined 6 measures for study: (1) ICV in milliliters, (2) total cortical gray matter (milliliters), (3) cerebral white matter volume (milliliters), (4) hippocampal volume (milliliters), (5) cortical surface area in centimeters squared, and (6) mean cortical thickness in millimeters. To further remove differences due to scanner type, MRI measures from the cohort of 3407 individuals that included those with medical exclusions were corrected using neuroHarmonize, a robust method for reducing machine-related differences in MRI data among individuals across a wide range of ages, adjusting for nonlinear differences using a generalized additive model^20,21,22^ (eMethods, eTable 1, and eFigure 1 in Supplement 1).

### Statistical Analysis

The goal of this study was to examine secular differences in various brain measures, including volume, area, and thickness. Given that secular differences in both intracranial and regional brain volumes may occur, regional volumes were not corrected for head size. Dependent variables included continuous measures of intracranial, cortical gray and white matter, and hippocampal volumes as well as cortical thickness and surface area. Birth decade, treated as an ordinal, categorical variable, was used as an independent predictor of secular differences in brain volumes, area, or thickness. Covariates included age and sex.

Least-squares regression with post hoc assessment of least-squares difference by Tukey honestly significant difference was used to examine secular trends and differences by birth decade. In addition, post hoc *t* test contrasts were used to determine differences between the reference birth decade of 1930 and other birth decades for the 6 measures. An additional analysis of differences based on a restricted sample of 1145 of similarly aged individuals born during the 1940s (mean [SD] age, 60 [2.8] years; range, 55-65 years) and 1950s (mean [SD] age, 59 [2.8] years; range, 55-65 years) was also performed to further disentangle MRI age from birth decade on the initial findings.

Given that the mean height of the population is increasing,^23^ we also examined the relationship between decade of birth, height, and ICV to estimate the degree to which ICV was similar to, or different from, overall progressive human skeletal growth. Initial analysis tested the association between ICV and decade of birth, adjusting for age and sex. Given that height and ICV are correlated, we also performed multivariable regression to investigate if ICV differed by decade of birth after adjusting for height.

## Results

### Sample Characteristics

**Figure 1.**
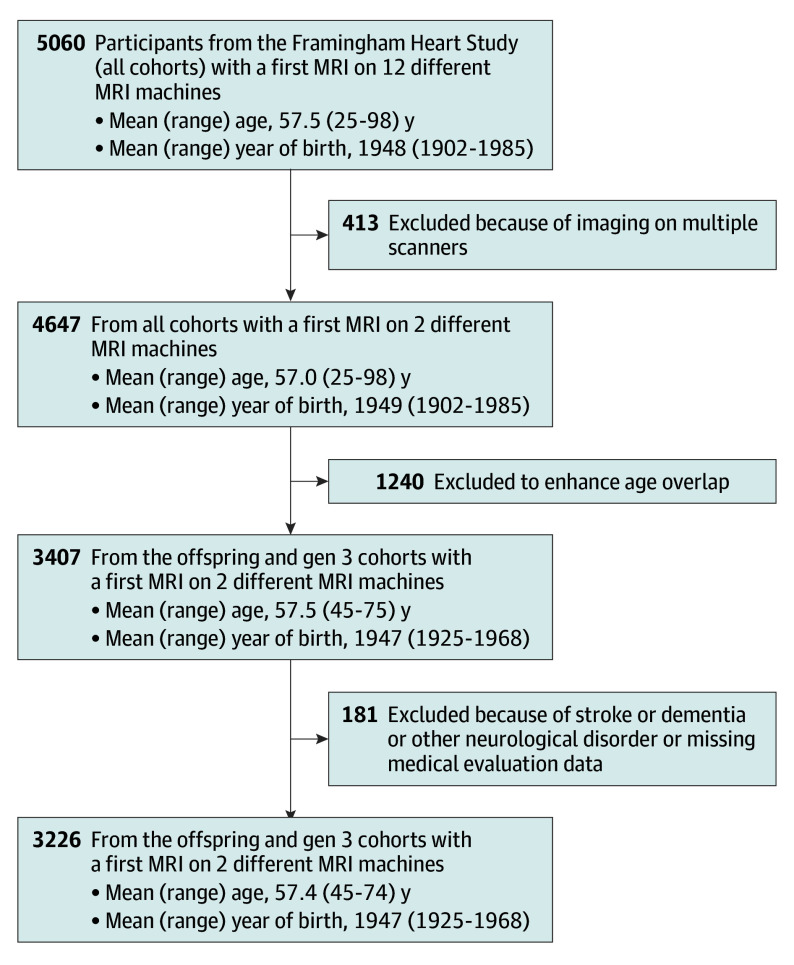
Sample Selection From 3 Framingham Heart Study Cohorts All individuals were included at the time of first magnetic resonance imaging (MRI). The initial group of participants that included members from all 3 cohorts were imaged on a total of 12 different MR machines, but more than 90% of participants were imaged on only 2 different scanners, leading to their selection as a subgroup. Further restriction to enhance age overlap resulted in a cohort of 3407 individuals from the offspring and third-generation (gen 3) cohorts, ranging in age at the time of their MRI from 45 to 74 years.

The final cohort sample consisted of 3226 individuals from the offspring and third-generation cohorts, of which 1706 (53%) were female and 1520 (47%) were male (Figure 1). The mean (SD) age at the time of the MRI was 57.4 (7.8 years) and ranged from 45 to 74 years (Table 1). Mean age at MRI differed significantly by decade of birth (Table 1), but considerable overlap in age occurred across various epochs. For example, individuals born in the 1950s varied in age from 45 to 71 years at time of their MRI, covering 86% of the age range, enabling sensitivity analysis between those born during the 1950s and those born during the 1940s with complete overlap of age distribution for the 2 cohorts. The specific distributions of age for various decades of birth for the primary and sensitivity analyses are summarized in eFigure 2 in Supplement 1.

**Table 1.** Demographics of Cohort by Decade of Birth

Characteristic	Birth decade					*P* value^a^
	1930s	1940s	1950s	1960s	1970s	
No. of participants	532	777	904	872	136	NA
Age, mean (SD) [range], y	69.7 (2.6) [65-74]	60.83 (3.8) [55-74]	55.3 (5.0) [45-71]	50.9 (3.1) [45-59]	45.7 (0.9) [45-48]	<.001
Sex, No. (%)						
Female	283 (53)	422 (54)	473 (52)	463 (53)	65 (48)	.70
Male	249 (47)	355 (46)	432 (48)	413 (47)	71 (52)	
Some college, No. (%)	162 (30)	300 (39)	468 (52)	449 (51)	85 (63)	<.001
*APOE* ε4 allele, No. (%)	116 (22)	171 (23)	204 (23)	171 (21)	30 (23)	.77
Height, mean (SD), in	66.0 (3.5)	66.3 (3.8)	67.0 (3.7)	67.3 (3.7)	67.5 (3.9)	<.001
Volume, mean (SD), mL						
ICV	1236 (123)	1259 (129)	1280 (123	1296 (128)	1317 (122)	<.001
Cortical gray matter	468.3 (45.5)	483.7 (47.5)	496.7 (47.8)	506.5 (49.1)	518.3 (47.1)	<.001
Hippocampus	6.42 (0.67)	6.70 (0.69)	6.84 (0.72)	6.86 (0.72)	6.85 (0.65)	<.001
Cortical thickness, mean (SD), mm	2.08 (0.17)	2.12 (0.18)	2.12 (0.16)	2.10 (0.16)	2.10 (0.14)	<.001
Cortical surface area, mean (SD), cm^2^	2056 (221)	2008 (235)	2030 (231)	2081 (224)	2104 (214)	<.001

Abbreviations: ICV, intracranial volume; MRI, magnetic resonance imaging; NA, not applicable.

^a^ One-way analysis of variance for continuous variables; χ^2^ test of independence for categorical variables.

The median decade of birth was the 1950s but ranged from 1930 to 1970, and almost half of the individuals (1464/3221 [46%]) achieved some college education. Significant differences by female sex at birth were found for height (−5.5 in), ICV (−155.0 mL), hippocampal volume (−0.64 mL), cortical surface area (−264 cm^2^), total cortical gray volume (−53.5 mL), and white matter volume (−63.3 mL).

### Impact of Decade of Birth

There was a significant secular trend for greater height (adjusting for age and sex) with mean (SD) height of 66.0 (3.5) in for individuals born during the 1930s compared with a mean (SD) height of 67.6 (3.9) in for individuals born during the 1970s.

**Figure 2.**
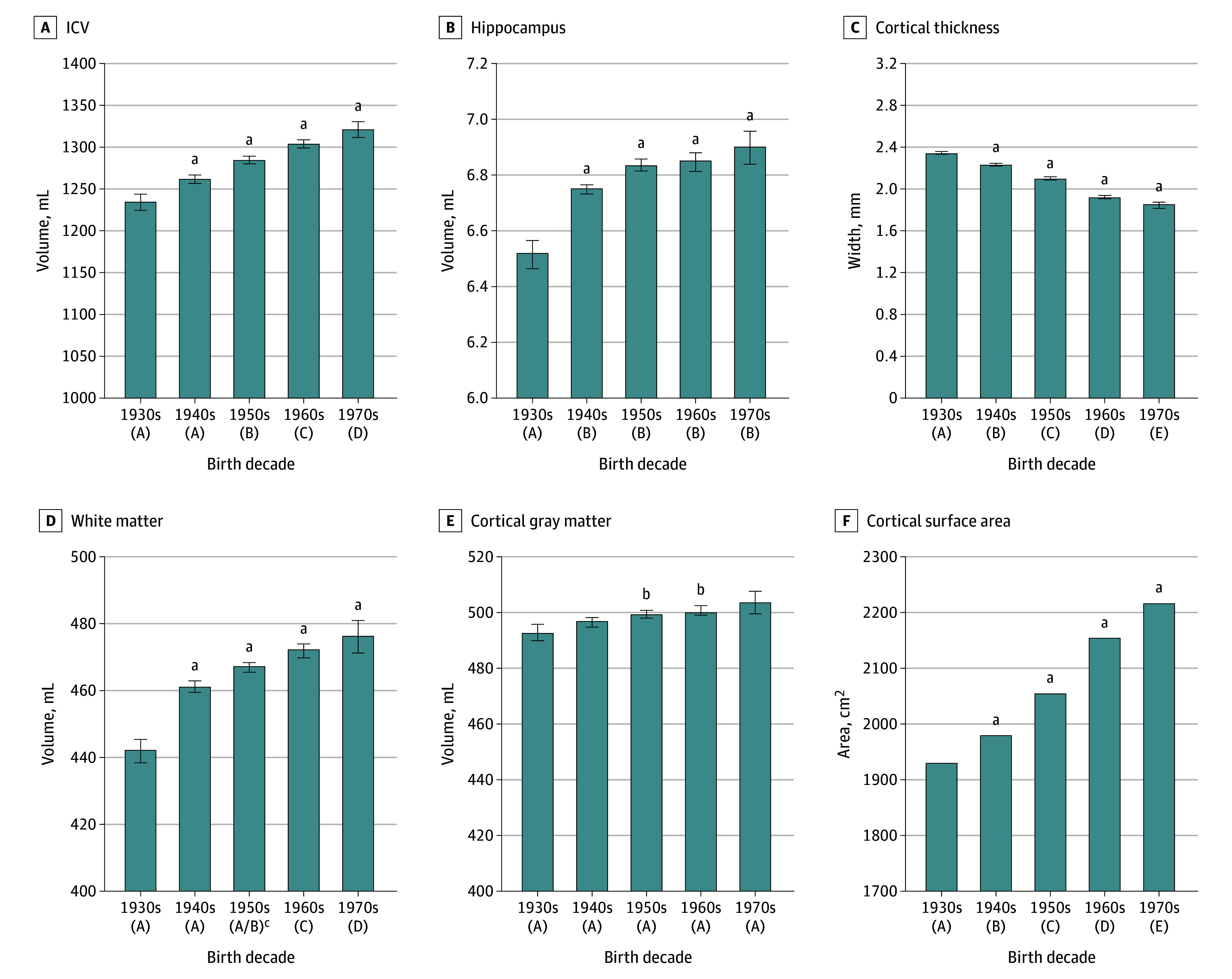
Least-Squares Mean Association (Adjusting for Age and Sex) Between Decade of Birth and Magnetic Resonance Imaging Measures The parenthetical letters A, B, C, D, and E under each decade indicate significant group differences using a Tukey honestly significant differences post hoc comparison. ICV indicates intracranial volume. ^a^*P* < .001 compared with 1930s. ^b^*P* < .05 compared with 1930s.

There was a secular trend for larger ICV (adjusting for age and sex) with an adjusted mean ICV of 1234 mL for individuals born during the 1930s and 1321 mL for individuals born in the 1970s (Figure 2 and Table 2). There was no sex × decade of birth interaction. Given the association between height and ICV, we further tested whether secular differences in ICV remained significant after adjusting for height. In a multivariable model that included adjustments for height, sex, and age, secular differences in ICV remained significant, varying from 1238 mL for those born during the 1930s to 1315 mL for those during the 1970s.

**Table 2.** Association of Birth Decade With MRI Measures by Brain Region in the Study Sample

Variable	LSM or Estimate (SE)^a^	95% CI	*P* value^b^
**ICV, mL**			
Trend	NA	NA	<.001
1930 Birth decade^c^	1234 (7.2)	NA	NA
Birth decades 1940-1930	30.0 (7.00)	15.99 to 43.28	<.001
Birth decades 1950-1940	21.2 (5.52)	10.34 to 31.97	<.001
Birth decades 1960-1950	19.2 (5.14)	9.084 to 29.25	<.001
Birth decades 1970-1960	17.1 (9.50)	−1.48 to 35.78	.07
Female sex	−77.1 (1.8)		<.001
Age at MRI	0.61 (0.47)	−0.31 to 1.524	.19
**Cortical gray matter, mL**			
Trend	NA	NA	.30
1930 Birth decade	492.9 (2.86)	NA	NA
Birth decades 1940-1930	3.75 (2.8)	−1.7 to 9.19	.18
Birth decades 1950-1940	3.01 (2.20)	−1.3 to 7.326	.17
Birth decades 1960-1950	1.78 (2.05)	−2.24 to 5.801	.39
Birth decades 1970-1960	2.81 (3.79)	−4.62 to 10.24	.46
Female sex	−26.5 (0.70)	−27.9 to −25.2	<.001
Age at MRI	−1.29 (0.186)	−1.66 to −0.93	<.001
**White matter, mL**			
Trend	NA	NA	<.001
1930 Birth decade	441.9 (3.4)	NA	NA
Birth decades 1940-1930	19.01 (3.3)	12.59 to 25.43	<.001
Birth decades 1950-1940	6.53 (2.59)	1.44 to 11.61	.01
Birth decades 1960-1950	4.89 (2.41)	0.151 to 9.637	.04
Birth decades 1970-1960	4.03 (4.46)	−4.73 to 12.8	.37
Female sex	−31.5 (0.82)	−33.2 to −29.9	<.001
Age at MRI	−0.88 (0.22)	−1.31 to −0.45	<.001
**Hippocampus, mL**			
Trend	NA	NA	<.001
1930 Birth decade	6.52 (0.05)	NA	NA
Birth decades 1940-1930	0.24 (0.04)	0.15 to 0.321	<.001
Birth decades 1950-1940	0.09 (0.03)	0.024 to 0.16	.008
Birth decades 1960-1950	0.01 (0.03)	−0.06 to 0.069	.86
Birth decades 1970-1960	0.05 (0.06)	−0.07 to 0.163	.45
Female sex	−0.32 (0.01)	−0.34 to −0.3	<.001
Age at MRI	−0.004 (0.003)	−0.01 to 0.001	.12
**Cortical thickness, mm**			
Trend	NA	NA	<.001
1930 Birth decade	2.14 (0.01)	NA	NA
Birth decades 1940-1930	−0.004 (0.014)	−0.13 to −0.08	<.001
Birth decades 1950-1940	−0.025 (0.009)	−0.15 to −0.11	<.001
Birth decades 1960-1950	−0.048 (0.009)	−0.19 to −0.16	<.001
Birth decades 1970-1960	−0.036 (0.017)	−0.11 to −0.05	<.001
Female sex	0.001 (0.003)	−5e-3 to 0.007	.72
Age at MRI	−0.015 (0.008)	−0.02 to −0.01	<.001
**Cortical surface area, cm^2^**			
Trend	NA	NA	<.001
1930 Birth decade	1933 (13)	NA	NA
Birth decades 1940-1930	49.3 (12.7)	24.38 to 74.24	.001
Birth decades 1950-1940	75.5 (10.1)	55.73 to 95.25	<.001
Birth decades 1960-1950	98.7 (9.4)	80.24 to 117.1	<.001
Birth decades 1970-1960	65.3 (17.4)	31.25 to 99.32	<.001
Female sex	−131 (3.2)	−137 to −124	<.001
Age at MRI	10.6 (0.85)	8.975 to 12.32	<.001

Abbreviations: ICV, intracranial volume; LSM least-squares mean; MRI, magnetic resonance imaging; NA, not applicable.

^a^ Regression β-weights.

^b^ *P* value for overall trend or *P* value for between-decade differences, female sex, and age. For example, 1940-1930 indicates the difference in brain measure between the LSM of the individuals born in the 1940 epoch minus the LSM of the individuals born in the 1930 epoch.

^c^ 1930 Birth decade denotes adjusted LSM estimate for that decade as anchor value for comparison.

Table 2 also summarizes the results of secular trend models for the regional MRI measures. There was a significant trend for greater volume associated with decade of birth for white matter and hippocampal volumes and cortical surface area. In contrast, decade of birth was significantly and negatively associated with cortical thickness. Figure 2 graphically summarizes these results. Differences across the birth decades (comparing the 1930 birth decade to the 1970 birth decade) accounted for a 6.6% greater volume (1234 mL; 95% CI, 1220-1248, vs 1321 mL; 95% CI, 1301-1341) for ICV, 7.7% greater volume (441.9 mL; 95% CI, 435.2-448.5, vs 476.3 mL; 95% CI, 467.0-485.7) for white matter, 2.2% greater volume (492.9 mL; 95% CI, 487.3-498.5, vs 504.3 mL; 95% CI, 496.3-512.2) for cortical gray matter, 5.7% greater value (6.51 mL; 95% CI, 6.42-6.60, vs 6.89 mL; 95% CI, 6.77-7.02) for hippocampal volume, and a 14.9% greater value (1933 cm^2^; 95% CI, 1908-1959, vs 2222 cm^2^; 95% CI, 2186-2259) for cortical surface area. However, cortical thickness was thinner by 20.9% (2.34 mm; 95% CI, 2.31-2.36, vs 1.85 mm; 95% CI, 1.81-1.88) over the same period. Sex-stratified analyses did not differ significantly from the primary analysis (eTable 2 and eFigure 3 in Supplement 1).

### Sensitivity Analyses

To minimize the influence of age at time of MRI on our findings, we performed an additional analysis further restricting age range and decade of birth to maximize age overlap. Table 3 summarizes the predicted values for the MRI volumes across 1145 individuals aged 55 to 65 years for those born during the 1940s as compared with those born during the 1950s. The mean (SD) age at MRI for those born in the 1940s was 60 (2.8) years, and for those born in the 1950s, it was 59 (2.8) years. Predicted volumes for those born in the 1950s remained greater than those born in the 1940s for ICV, hippocampal volume, and cortical surface area. Cortical thickness was significantly less, confirming the results noted with the larger cohort. Differences in size between the birth decades were 1.7% greater for ICV, 0.2% greater for cortical gray matter, 0.1% greater for white matter, 1.3% greater for hippocampal volume, and 5% greater for cortical surface area. However, cortical thickness was thinner by 17%. Graphic representation of the findings can be found in eFigure 4 in Supplement 1.

**Table 3.** Association of Birth Decade With MRI Measures by Brain Region in the Restricted Sample of 1145 Individuals

Variable	Estimate (SE)^a^	*P* value^b^
**ICV, mL**		
1940 Birth decade^c^	1269 (3.8)	NA
1950 Birth decade	1290 (4.8)	
Difference	21.2 (6.2)	<.001
Female sex	−79.8 (2.9)	<.001
Age at MRI	0.04 (1.0)	.97
**Cortical gray, mL**		
1940 Birth decade	495.8 (1.5)	NA
1950 Birth decade	496.9 (1.9)	
Difference	1.12 (2.4)	.65
Female sex	−27.0 (1.2)	<.001
Age at MRI	1.28 (0.4)	.002
**White matter, mL**		
1940 Birth decade	462 (1.8)	NA
1950 Birth decade	465 (2.3)	
Difference	3.6 (2.9)	.22
Female sex	−32.1 (1.4)	<.001
Age at MRI	1.57 (0.5)	.002
**Hippocampus, mL**		
1940 Birth decade	6.76 (0.02)	NA
1950 Birth decade	6.85 (0.03)	
Difference	0.09 (0.04)	.02
Female sex	−0.33 (0.02)	<.001
Age at MRI	−0.013	.05
**Cortical thickness, mm**		
1940 Birth decade	2.20 (0.007)	NA
1950 Birth decade	2.03 (0.009)	
Difference	−0.17 (0.011)	<.001
Female sex	−0.003 (0.005)	.57
Age at MRI	−0.01 (0.001)	<.001
**Cortical surface area, cm^2^**		
1940 Birth decade	2005 (7.0)	NA
1950 Birth decade	2102 (8.9)	
Difference	97.7 (11.4)	<.001
Female sex	−138 (5.4)	<.001
Age at MRI	10.4 (1.9)	<.001

Abbreviations: ICV, intracranial volume; MRI, magnetic resonance imaging; NA, not applicable.

^a^ Regression β-weights.

^b^ *P* value for between-decade differences, female sex, and age.

^c^ There were 706 participants in the 1940 birth epoch and 439 in the 1950 birth epoch.

A second sensitivity analysis examined the effect of excluding individuals with medical conditions. Inclusion of individuals with medical conditions did not alter the significance of the results (data not shown).

## Discussion

This study found significant secular differences in larger volumes of the cranium (ICV), cortical white matter, hippocampus, and cortical surface area with progressive decades of birth. Secular differences in ICV also remained significant after adjusting for height. We hypothesize that larger brain volumes indicate larger brain development and potentially greater “brain reserve”^24,25,26,27,28^ that could explain the declining incidence of dementia as previously reported.^5^

Confounding by association of age at MRI with decade of birth was addressed through a sensitivity analysis. Results from this secondary analysis found that restriction of the age of participants to 55 to 65 years and being born during the 1940s and 1950s resulted in a cohort with considerable overlap in age distribution and minimal age-related differences in brain volumes or thickness. Results of this secondary analysis, while reduced in magnitude, confirmed larger ICV and hippocampal volumes as well as cortical surface area associated with the 1950 birth decade (Table 2 and eFigure 4 in Supplement 1).

Analysis of the entire cohort resulted in multiple unique findings. First, we noted that ICV volume was greater with birth decade even when adjusting for height, sex, and age, and this effect did not vary by sex. These results suggest that ICV is likely increasing through secular trends either in processes different from those influencing height^23^ or the magnitudes of influence on these processes. Second, although birth decade was used as a measure for secular differences, post hoc analyses using Tukey honestly significant differences found that the largest secular effects for ICV, white matter volume, and hippocampal volume occurred between the 1930 and 1940 epochs (Figure 2 and Table 2). Conversely, cortical thickness was less at each epoch. Further analyses found that less cortical thickness coincided with larger ICV, cerebral white matter volume, and cortical surface area and only limited change in cortical volume.

Rakic^29^ described how the radial unit lineage model would be consistent with our findings. In this model, expansion of the cerebral cortex occurs through increasing convolutions and expanding surface area while limiting change in cortical thickness. White et al^30^ extended this concept to “gyrification” of the cerebral cortex, which they noted results in a surface area “1700 times larger [in humans] than in shrews, yet the thickness of the cortex is only 6 times greater.” Like Rakic,^29^ White et al^30^ emphasized the computational utility of expanding surface area over thickness that allows for “an optimized compaction of neuronal fibers with an efficient transit time for neuronal signaling.” Consequently, the larger cortical white matter volumes found with progressive decades of birth may reflect the increased cortical surface area through increased neuronal fibers resulting from increased gyrification. Accordingly, the increased gyrification (in the absence of significant increase in cortical gray matter volume) leads to reductions in cortical thickness, as seen in our data. Importantly, both Rakic^29^ and White et al^30^ emphasized regional differences in gyrification are likely under unique genetic influence.^31^ Such an analysis is beyond the scope of this report but could add further information regarding the biology of reduction in cortical thickness, as seen with our more global measures.

How might these secular effects modify the likelihood of later life dementia? Brain growth begins in utero, increases throughout childhood, and reaches a maximum size in early adulthood.^32,33,34,35^ ICV is highly associated with brain growth during normal development,^27^ whereas aging or disease-related brain-volume decreases do not alter ICV. Thus, adult ICV is a stable and valid measure for maximal attained brain size, widely used as a proxy for brain reserve,^36,37^ and is an important predictor of cognition in old age.^28^ Conversely, cross-sectional, and longitudinal age-related differences in brain volume measures are associated with cognitive performance in aging and disease.^38,39,40^ Hippocampal volume loss, in particular, is considered to be sensitive to early degenerative diseases such as Alzheimer disease.^41,42^ Although absolute volumes are not associated with cognitive ability per se, as illustrated by the fact that the women in this study had significantly smaller hippocampal volumes, but multiple studies show that cognitively normal women outperform men on tests of episodic memory.^43^ However, loss of brain tissue within an individual is strongly indicative of pathological effects,^44^ which therefore may be buffered by larger structural brain development. Alternatively, larger structural brain development may be a surrogate for other processes ongoing during development and early adult life, such as increased brain connectivity.^45^ Increased connectivity is consistent with the radial unit lineage hypothesis^29^ that enables increased neuronal connectivity through cortical expansion and gyrification.^30^ Increased connectivity could explain our finding of increased white matter volume, could mitigate the impact of age-related diseases on cognitive performance, and fits well with the scaffolding hypothesis of cognitive reserve.^46^

While ICV and brain size are under substantial genetic influence,^31,47,48,49,50^ the timeline of effect found with our results indicates that early life environmental influences are more likely contributors. Life course perspectives emphasize the impact of early life experiences on brain health^51,52^ that also translate into larger brain structures^53^ and reduced risk for later-life dementia through improved reserve.^54^ Similarly, efforts to improve cardiovascular health during adulthood^55,56,57^ that occurred over the time duration of this study^58^ are associated with reduced incidence of cognitive impairment^59^ and dementia,^60^ indicating that modifying these factors could also serve to improve resistance to late-life dementia.^61^

In summary, our results indicate that ICV, white matter volume, and hippocampal volume as well as cortical surface area have increased over decades of birth ranging from 1930 to 1970. Differences were also found for a select, relatively age-matched subgroup born in the 1940s and 1950s. These findings likely reflect both secular improvements in early life environmental influences through health, social-cultural, and educational factors,^53^ as well as secular improvements in modifiable dementia risk factors leading to better “brain health” and reserve.^58^ While these effects are likely to be small at the level of the individual, they are likely to be substantial at the population level, adding to growing literature that suggests optimized brain development and ideal health through modification of risk factors could substantially modify the effect of common neurodegenerative diseases such as stroke and Alzheimer disease on dementia incidence.^6,56,62^ Moreover, taken together with increases in IQ throughout the 20th century,^63^ these secular trends in brain volume may contribute to greater cognitive resilience.

### Strengths and Limitations

The strengths of our study include the design of the FHS, which began in 1948 and has followed a community of individuals with comprehensive health evaluations throughout much of their life span. The addition of MRI beginning in 1999 enabled quantitative brain assessment across all 3 generations, spanning more than 80 years’ difference in dates of birth. Large sample size across decades of birth also enabled reliable sensitivity analyses that might not be accomplished with smaller studies. The duration of observation that includes younger individuals also suggests that this secular trend may be continuing. Finally, the fact that more than 90% of participants were imaged on just 2 MRI machines also helped reduce the effect machine differences, which were further reduced using the neuroHarmonize statistical harmonization method.

This study is not without limitations. First, and most importantly, the FHS cohort is predominately non-Hispanic White, healthy, and well educated and therefore not representative of the broader US population. For example, current evidence indicates that social-cultural^53,64,65,66^ and health disparities,^2^ which are more common among non-White individuals^58^ in the US, may adversely affect brain health. Second, this is a cross-sectional study that has limited causal inference. Longitudinal analyses showing secular differences in rates of regional brain atrophy would further support evidence of increased brain reserve through resilience to age-related atrophy.^26^

## Conclusions

This study extends current knowledge by showing that secular trends in brain structure are occurring. The larger brain structure, which may reflect improved brain development and brain health, is at least 1 manifestation of improved brain reserve that could buffer the effect of late-life diseases on incident dementia.

## Notes

Supplement 1. **eMethods.** neuroHarmonize MR machine harmonization **eTable 1.** Application of neuroHarmonize to the data for this study **eFigure 1.** Uncorrected versus neuroHarmonized corrected normalized residuals (age and sex) **eFigure 2.** Summary of cohort age distribution by birth decade **eFigure 3.** Sex stratified regional analyses **eTable 2.** Sex stratified regional analyses **eFigure 4.** Predicted group differences from multiple regression
eReferences

Supplement 2. Data sharing statement
