# Supplementary material for: Trends in Intracranial and Cerebral Volumes of Framingham Heart Study Participants Born 1930 to 1970
Source: JAMA Neurol. 2024 Mar 25;81(5):471–80. doi: 10.1001/jamaneurol.2024.0469 (PMC10964161; doi:10.1001/jamaneurol.2024.0469)
Supplement: Supplement 1. — eMethods. neuroHarmonize MR machine harmonization eTable 1. Application of neuroHarmonize to the data for this study eFigure 1. Uncorrected versus neuroHarmonized corrected normalized residuals (age and sex) eFigure 2. Summary of cohort age distribution by birth decade eFigure 3. Sex stratified regional analyses eTable 2. Sex stratified regional analyses eFigure 4. Predicted group differences from multiple regression eReferences [file jamaneurol-e240469-s001.pdf]

# Supplemental Online Content

DeCarli C, Maillard P, Pase MP, et al. Trends in intracranial and cerebral volumes of Framingham Heart Study participants born 1930 to 1970. *JAMA Neurol*. Published online March 25, 2024. doi:10.1001/jamaneurol.2024.0469

**eMethods.** neuroHarmonize MR machine harmonization

**eTable 1.** Application of neuroHarmonize to the data for this study

**eFigure 1.** Uncorrected versus neuroHarmonized corrected normalized residuals (age and sex)

**eFigure 2.** Summary of cohort age distribution by birth decade

**eFigure 3.** Sex stratified regional analyses

**eTable 2.** Sex stratified regional analyses

**eFigure 4.** Predicted group differences from multiple regression

**eReferences**

This supplemental material has been provided by the authors to give readers additional information about their work.

© 2024 DeCarli C et al. *JAMA Neurology.*

eMethods. neuroHarmonize MR Machine Harmonization

Confounding of biological processes by MR machine induced bias is a common and recognized problem in quantitative human brain imaging. A variety of approaches exist, but ComBat, a method developed to adjust for batch effects in microarray expression data[1] is proving to be robust at reducing machine related differences in MRI data[2-4]. Additional work has extended the application of ComBat to groups of individuals across the span of human aging using a generalized additive model (GAM)[4]. ComBat-GAM (aka, neuroHarmonize) was applied to the MRI data used in this example, adjusting for relevant covariates of age at MRI and sex as prescribed by the method developers[4].

neuroHarmonize extends the functionality of neuroCombat [3] which is hosted on GitHub: https://github.com/ncullen93/neuroCombat and is hosted on PYPI as neuroHarmonize 2.1.0. Application of neuroHarmonize to the data used for this study is summarized in eTable 1. Comparison of z-normalized residuals (age and sex) for uncorrected and corrected regions is graphically illustrated in eFigure 1.

eTable 1.

| | | Uncorrected | | | Corrected | | |
|---|---|---|---|---|---|---|---|
| **Region** | Regressor | Beta | Std Error | P value | Beta | Std Error | P value |
| **ICV** | MRI Age | -1.4867 | 0.2505 | 0.0000 | -1.4888 | 0.2504 | 0.0000 |
| **ICV** | Sex[Female] | -77.2109 | 1.7576 | 0.0000 | -77.1909 | 1.7566 | 0.0000 |
| **ICV** | MRI Machine[Avanto] | 13.6980 | 1.9500 | 0.0000 | 0.2484 | 1.9489 | 0.8986 |
| **White Matter** | MRI Age | -1.8074 | 0.1182 | 0.0000 | -1.8067 | 0.1182 | 0.0000 |
| **White Matter** | Sex[Female] | -31.5587 | 0.8293 | 0.0000 | -31.5622 | 0.8290 | 0.0000 |
| **White Matter** | MRI Machine[Avanto] | -0.3414 | 0.9201 | 0.7106 | -0.0327 | 0.9198 | 0.9717 |
| **Hippocampus** | MRI Age | -0.0150 | 0.0016 | 0.0000 | -0.0150 | 0.0016 | 0.0000 |
| **Hippocampus** | Sex[Female] | -0.3200 | 0.0111 | 0.0000 | -0.3201 | 0.0111 | 0.0000 |
| **Hippocampus** | MRI Machine[Avanto] | 0.0255 | 0.0123 | 0.0383 | 0.0003 | 0.0123 | 0.9789 |
| **Cortical Gray** | MRI Age | -1.5576 | 0.0999 | 0.0000 | -1.5576 | 0.0999 | 0.0000 |
| **Cortical Gray** | Sex[Female] | -26.5363 | 0.7009 | 0.0000 | -26.5318 | 0.7006 | 0.0000 |
| **Cortical Gray** | MRI Machine[Avanto] | 4.8483 | 0.7777 | 0.0000 | 0.0876 | 0.7773 | 0.9103 |
| **Cortical Thickness** | MRI Age | -0.0030 | 0.0004 | 0.0000 | -0.0028 | 0.0004 | 0.0000 |
| **Cortical Thickness** | Sex[Female] | 0.0007 | 0.0030 | 0.8191 | 0.0013 | 0.0029 | 0.6596 |
| **Cortical Thickness** | MRI Machine[Avanto] | -0.0547 | 0.0033 | 0.0000 | -0.0009 | 0.0033 | 0.7849 |

© 2024 DeCarli C et al. *JAMA Neurology.*

eFigure 1. Uncorrected versus neuroHarmonized corrected normalized residuals (age and sex). Although the magnitude of differences is small, uncorrected regions show subtle variation that is removed with correction, particularly for cortical thickness.

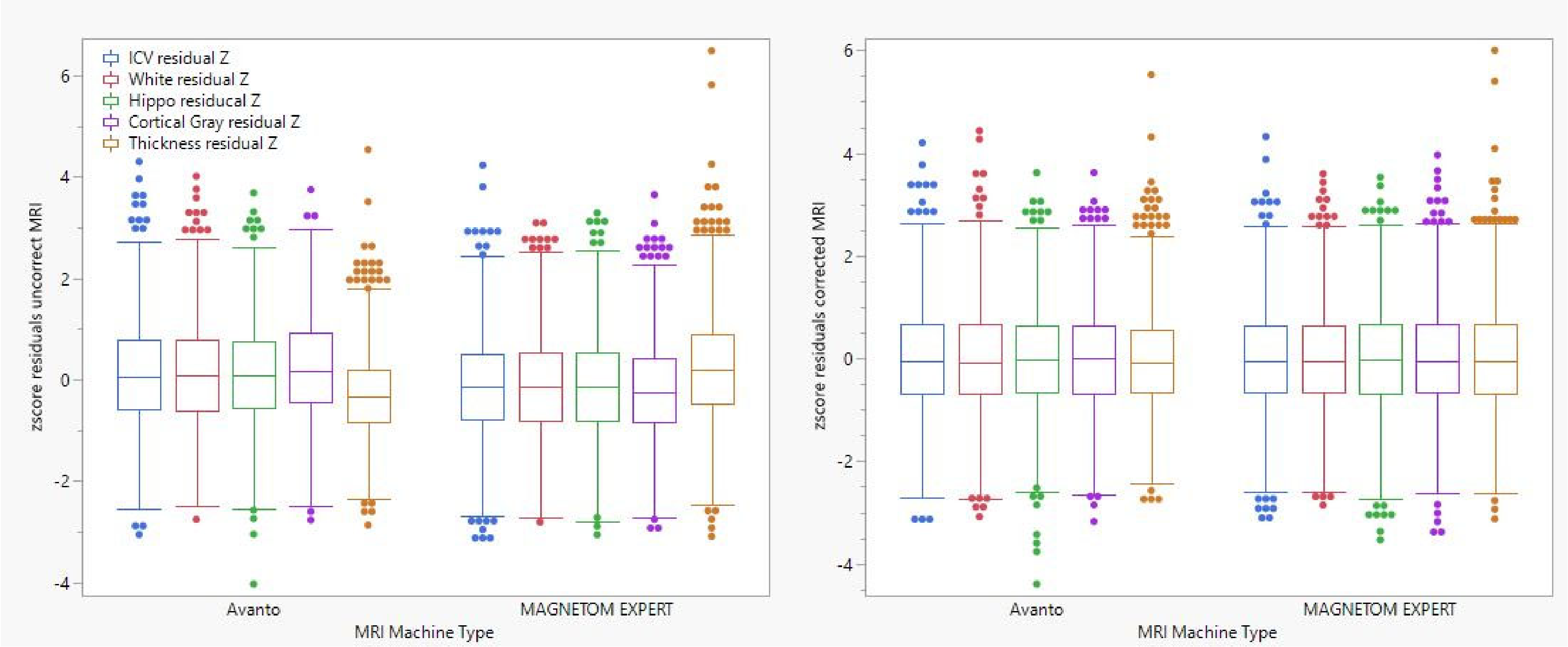


© 2024 DeCarli C et al. *JAMA Neurology.*

Study Cohort and Sensitivity Analysis age distributions by decade of birth

eFigure 2. Summary of cohort age distribution by birth decade for the initial analysis (2a) and differences with selective age restriction used for sensitivity analysis (2b).

eFigure 2a. Age distribution of original study cohort

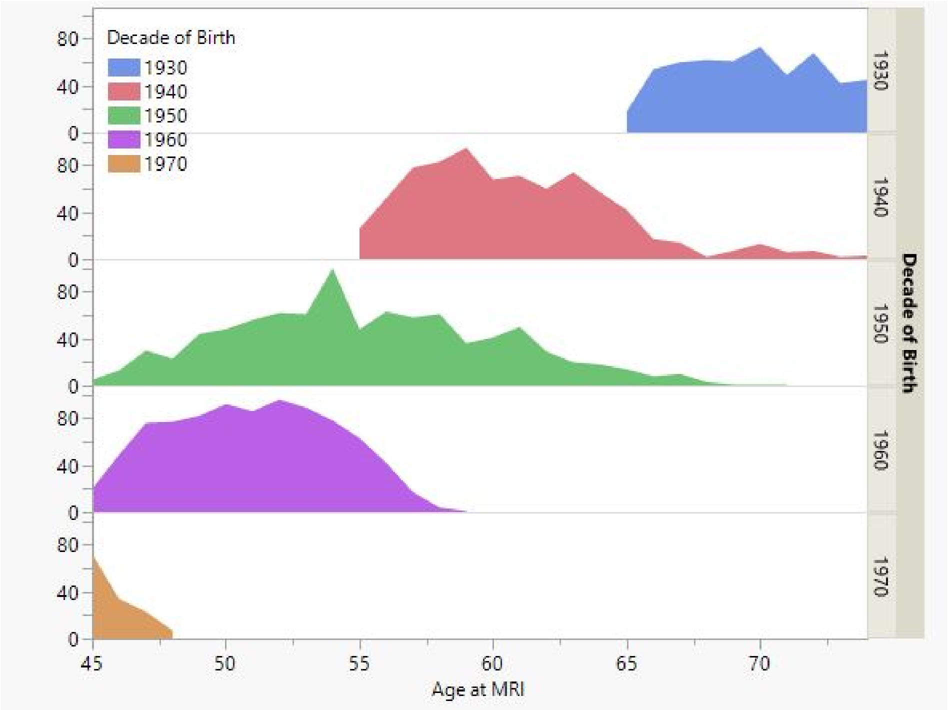


.

© 2024 DeCarli C et al. *JAMA Neurology.*

eFigure 2b. Age distribution of select cohort used for sensitivity analysis.

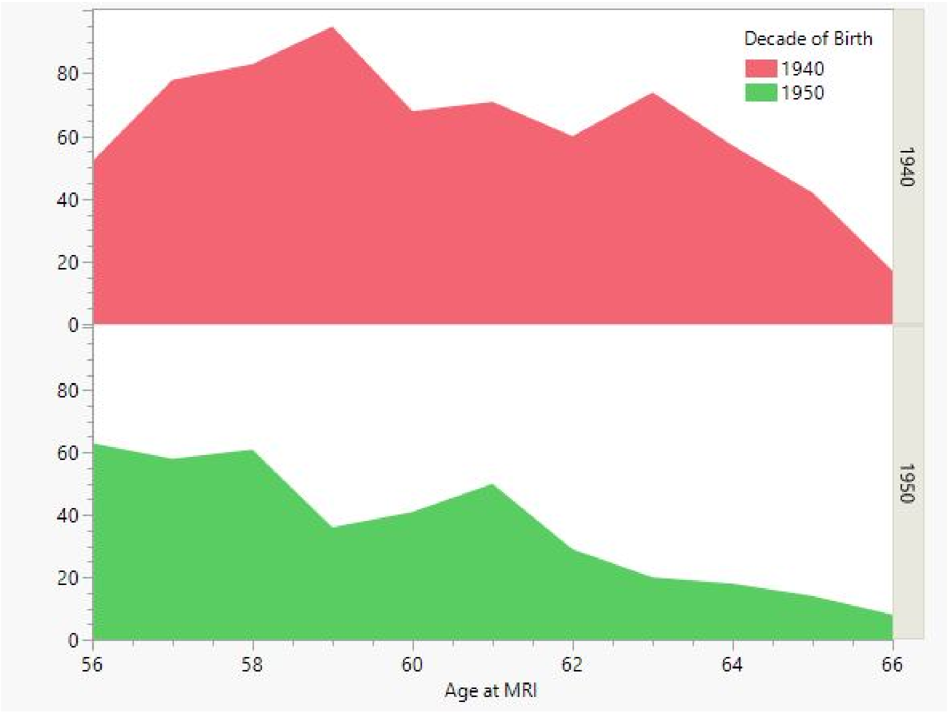


© 2024 DeCarli C et al. *JAMA Neurology.*

eFigure 3.  Sex Stratified Regional Analyses

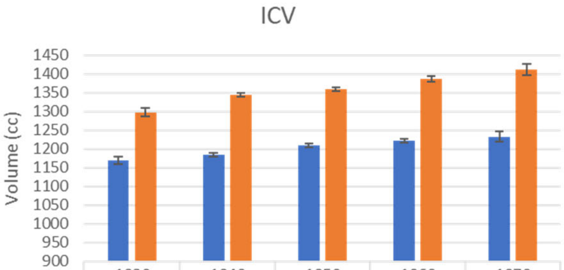


| | 1930 | 1940 | 1950 | 1960 | 1970 |
|---|---|---|---|---|---|
| Female | 1169 | 1184 | 1210 | 1222 | 1233 |
| Male | 1297 | 1344 | 1360 | 1387 | 1411 |

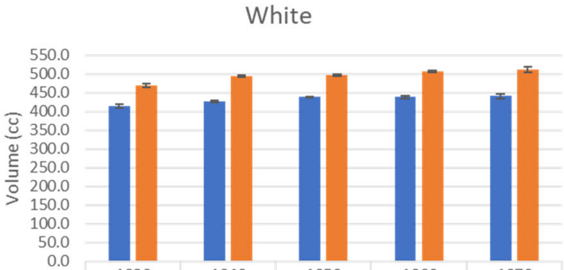


| | 1930 | 1940 | 1950 | 1960 | 1970 |
|---|---|---|---|---|---|
| Female | 414.8 | 427.3 | 438.1 | 438.0 | 440.9 |
| Male | 468.3 | 494.8 | 496.5 | 506.9 | 511.1 |

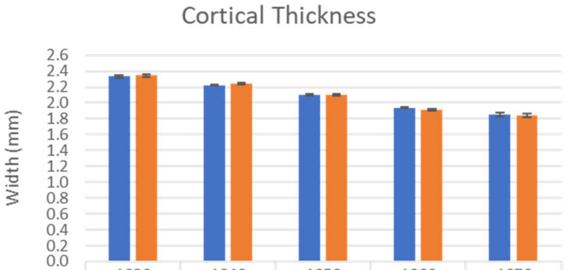


| | 1930 | 1940 | 1950 | 1960 | 1970 |
|---|---|---|---|---|---|
| Female | 2.33 | 2.22 | 2.10 | 1.94 | 1.85 |
| Male | 2.34 | 2.24 | 2.10 | 1.91 | 1.84 |

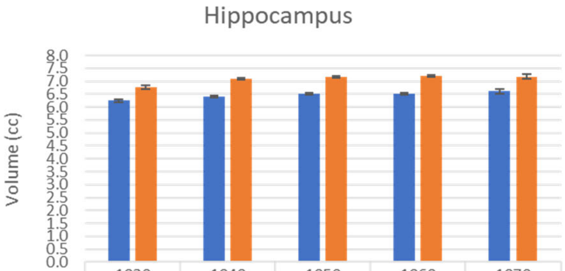


| | 1930 | 1940 | 1950 | 1960 | 1970 |
|---|---|---|---|---|---|
| Female | 6.25 | 6.42 | 6.52 | 6.50 | 6.61 |
| Male | 6.77 | 7.09 | 7.17 | 7.20 | 7.18 |

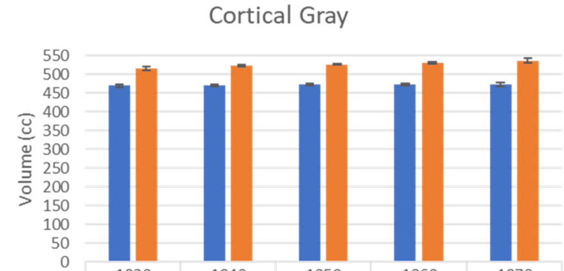


| | 1930 | 1940 | 1950 | 1960 | 1970 |
|---|---|---|---|---|---|
| Female | 470.3 | 469.8 | 473.1 | 473.5 | 472.7 |
| Male | 515.0 | 523.5 | 526.2 | 529.6 | 535.7 |

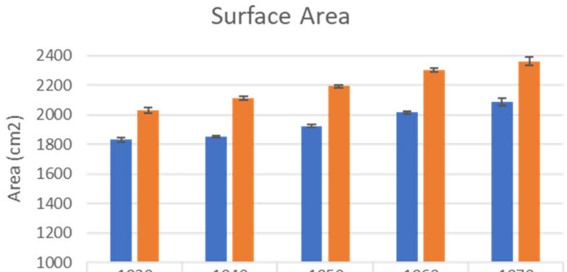


| | 1930 | 1940 | 1950 | 1960 | 1970 |
|---|---|---|---|---|---|
| Female | 1831 | 1852 | 1924 | 2015 | 2086 |
| Male | 2032 | 2113 | 2193 | 2301 | 2363 |

© 2024 DeCarli C et al. *JAMA Neurology.*

eTable 2.  Sex Stratified Regional Analyses

| Region | Sex | Estimator Variable | P value for trend |
|---|---|---|---|
| ICV | Female | Age at MRI | 0.9742 |
| ICV | Female | Birth Decade | **0.0003** |
| ICV | Male | Age at MRI | 0.0674 |
| ICV | Male | Birth Decade | **<.0001** |
| White Matter | Female | Age at MRI | 0.0043 |
| White Matter | Female | Birth Decade | **0.0005** |
| White Matter | Male | Age at MRI | 0.0047 |
| White Matter | Male | Birth Decade | **<.0001** |
| Cortical Gray | Female | Age at MRI | <.0001 |
| Cortical Gray | Female | Birth Decade | 0.8338 |
| Cortical Gray | Male | Age at MRI | <.0001 |
| Cortical Gray | Male | Birth Decade | 0.1802 |
| Hippocampus | Female | Age at MRI | 0.1408 |
| Hippocampus | Female | Birth Decade | **0.0051** |
| Hippocampus | Male | Age at MRI | 0.4204 |
| Hippocampus | Male | Birth Decade | <**.0001** |
| Cortical Thickness | Female | Age at MRI | <.0001 |
| Cortical Thickness | Female | Birth Decade | **<.0001** |
| Cortical Thickness | Male | Age at MRI | <.0001 |
| Cortical Thickness | Male | Birth Decade | **<.0001** |
| Surface Area | Female | Age at MRI | <.0001 |
| Surface Area | Female | Birth Decade | **<.0001** |
| Surface Area | Male | Age at MRI | <.0001 |
| Surface Area | Male | Birth Decade | **<.0001** |

Values in **bold** indicate significant trends by Birth Decade.

© 2024 DeCarli C et al. *JAMA Neurology.*

eFigure 4. Predicted group differences from multiple regression that included predictors of birth decade, sex, and age, superimposed on raw volume, area, or thickness data for each region by sex to better illustrate secular differences.

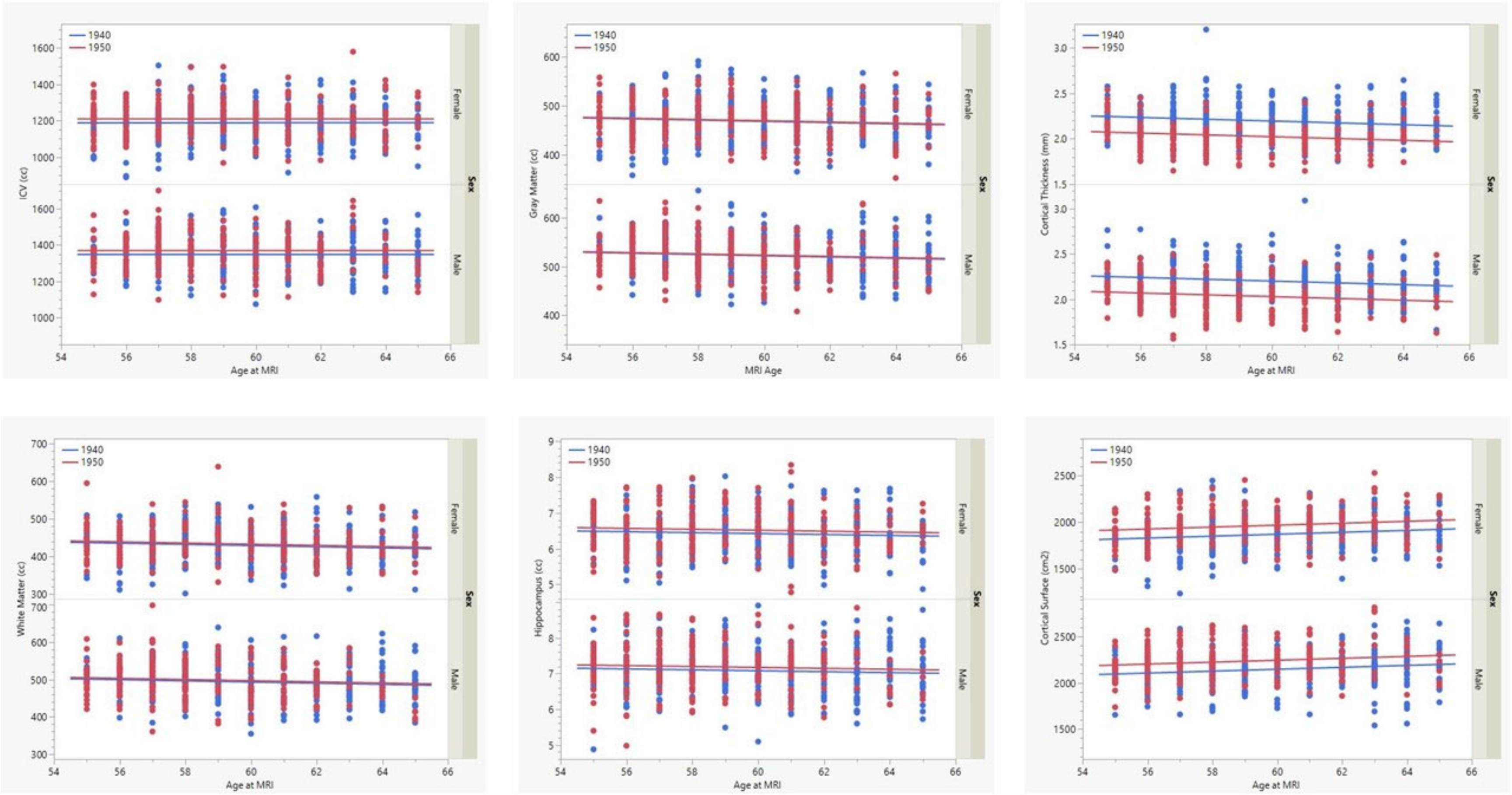


© 2024 DeCarli C et al. *JAMA Neurology.*

eReferences

1. Johnson WE, Li C, Rabinovic A. Adjusting batch effects in microarray expression data using empirical Bayes methods. Biostatistics 2007;8:118-127.
2. Fortin JP, Parker D, Tunc B, et al. Harmonization of multi-site diffusion tensor imaging data. Neuroimage 2017;161:149-170.
3. Fortin JP, Cullen N, Sheline YI, et al. Harmonization of cortical thickness measurements across scanners and sites. Neuroimage 2018;167:104-120.
4. Pomponio R, Erus G, Habes M, et al. Harmonization of large MRI datasets for the analysis of brain imaging patterns throughout the lifespan. Neuroimage 2020;208:116450.

© 2024 DeCarli C et al. *JAMA Neurology.*
