# Supplementary material for: Trends in Intracranial and Cerebral Volumes of Framingham Heart Study Participants Born 1930 to 1970
Source: JAMA Neurol. 2024 Mar 25;81(5):471–80. doi: 10.1001/jamaneurol.2024.0469 (PMC10964161; doi:10.1001/jamaneurol.2024.0469)
Supplement: Supplement 2. — Data sharing statement [file jamaneurol-e240469-s002.pdf]

Data Sharing Statement

DeCarli. Trends in Intracranial and Cerebral Volumes of Framingham Heart Study Participants Born 1930 to 1970. *JAMA Neurol*. Published March 25, 2024. doi:10.1001/jamaneurol.2024.0469

Data
Data available: No

Additional Information
Explanation for why data not available: Data are made available through Db Gap as stated in the data sharing section.
